# Characterization of primary models of human trophoblast

**DOI:** 10.1242/dev.199749

**Published:** 2021-11-05

**Authors:** Megan A. Sheridan, Xiaohui Zhao, Ridma C. Fernando, Lucy Gardner, Vicente Perez-Garcia, Qian Li, Steven G. E. Marsh, Russell Hamilton, Ashley Moffett, Margherita Y. Turco

**Affiliations:** 1 Department of Pathology, University of Cambridge, Cambridge CB2 1QP, UK; 2 Centre for Trophoblast Research, University of Cambridge, Cambridge CB2 3EG, UK; 3 Department of Physiology, Neuroscience and Development, University of Cambridge, Cambridge CB2 3EG, UK; 4 Centro de Investigación Príncipe Felipe, Eduardo Primo Yúfera, Valencia 46012, Spain; 5 Department of Genetics, University of Cambridge, Cambridge CB2 3EH, UK; 6 Anthony Nolan Research Institute, Royal Free Hospital, London NW3 2QG, UK; 7 UCL Cancer Institute, Royal Free Campus, London WC1E 6DD, UK

**Keywords:** HLA, Trophoblast organoids, Trophoblast stem cells, Models, Primary cells

## Abstract

Two recently developed models, trophoblast organoids and trophoblast stem cells (TSCs), are useful tools to further the understanding of human placental development. Both differentiate from villous cytotrophoblast (VCT) to either extravillous trophoblast (EVT) or syncytiotrophoblast (SCT). Here, we compare the transcriptomes and miRNA profiles of these models to identify which trophoblast they resemble *in vivo*. Our findings indicate that TSCs do not readily undergo SCT differentiation and closely resemble cells at the base of the cell columns from where EVT derives. In contrast, organoids are similar to VCT and undergo spontaneous SCT differentiation. A defining feature of human trophoblast is that VCT and SCT are human leukocyte antigen (HLA) null, whereas EVT expresses HLA-C, -G and -E molecules. We find that trophoblast organoids retain these *in vivo* characteristics. In contrast, TSCs express classical HLA-A and HLA-B molecules, and maintain their expression after EVT differentiation, with upregulation of HLA-G. Furthermore, HLA expression in TSCs differs when grown in 3D rather than in 2D, suggesting that mechanical cues are important. Our results can be used to select the most suitable model for the study of trophoblast development, function and pathology.

## Abstract (precis)

**Summary:** Characterization of trophoblast organoids and trophoblast stem cells as exciting models of human placentation enables the selection of the most suitable system to address specific research questions.

## INTRODUCTION

The placenta has many biological functions that support the developing fetus. Trophoblasts, the main cell type of the placenta, arise from the trophectoderm of the implanting blastocyst. After implantation, trophoblast differentiates along two main pathways: villous and extravillous. Placental villi are surrounded by a layer of villous cytotrophoblast (VCT), which fuses to form a multi-nucleated layer of syncytiotrophoblast (SCT). This syncytium is in direct contact with maternal blood and is the principal site of nutrient and oxygen exchange. Where villi make contact with the maternal decidua at the tips of anchoring villi, VCT proliferates to form cell columns. A possible location for trophoblast progenitors is at the base of these cell columns (the ‘column niche’), marked by ITGA2/NOTCH1 expression (Haider et al., 2016; Lee et al., 2018). At their distal ends, the columns merge to form the cytotrophoblastic shell, which initially encapsulates the conceptus at the maternal-fetal interface. As the trophoblast migrates away from the progenitor niche at the base of the column, it undergoes a process similar to epithelial-to-mesenchymal transition and differentiate to extravillous trophoblast (EVT) (Apps et al., 2011; Davies et al., 2016). Interstitial EVT invades through the decidual stroma to remodel the maternal spiral arteries and migrates as far as the myometrium, where they fuse into placental bed giant cells (Boyd and Hamilton, 1970). Interstitial EVT interacts with maternal decidual immune and stromal cells. In addition, endovascular EVT migrates down the inside of the spiral arteries and transiently replace the endothelium.

Given ethical and logistical limitations, little is known about human placental development and trophoblast differentiation. Recently, several groups have described the propagation and differentiation of primary trophoblast *in vitro* in a two-dimensional (2D) stem cell model (Okae et al., 2018) and a three-dimensional (3D) trophoblast organoid (TO) model (Haider et al., 2018; Turco et al., 2018). Human trophoblast stem cells (TSCs) are isolated from outgrowths of blastocysts or from first-trimester placentas. They can be cultured long term or stimulated to differentiate into either SCT or EVT. Trophoblast organoids are also derived from first-trimester placentas and form villous-like structures, composed of proliferative VCT with spontaneous differentiation to multi-nucleated SCT. Altering the culture conditions promotes differentiation to EVT. Both of these models are reported to meet the following criteria characterizing first-trimester trophoblast *in vivo* with: (1) expression of typical trophoblast markers; (2) a distinctive profile of human leukocyte antigen (HLA) class I molecules; (3) expression of microRNAs (miRNAs) from the chromosome 19 cluster (C19MC); and (4) methylation of the *ELF5* promoter (Lee et al., 2016).

Interaction of the allogeneic placenta with maternal immune cells occurs at the site of placentation in the decidua basalis. Human trophoblast has a unique HLA expression profile (Faulk and Temple, 1976; Ellis et al., 1986; Kovats et al., 1990; King et al., 1996, 2000; Apps et al., 2009; Hackmon et al., 2017). Yet, how HLA expression is regulated in trophoblast is still under investigation (Tilburgs et al., 2017; Johnson et al., 2018; Reches et al., 2020; Li et al., 2021). Neither VCT nor SCT expresses any HLA class I or class II molecules. There are six HLA class I loci, defined as either classical (HLA-A, HLA-B and HLA-C) or nonclassical (HLA-E, HLA-F and HLA-G). EVT only expresses nonclassical HLA-E and -G and classical HLA-C, and the polymorphic class I molecules, HLA-A and HLA-B, are not expressed (Hutter et al., 1996; Moffett and Loke, 2006; Apps et al., 2009). This distinctive pattern of trophoblast HLA expression has been particularly useful in defining the identity of *in vitro* trophoblast models (Lee et al., 2016) and is also important for recognition of the fetus by uterine natural killer (NK) cells during pregnancy (Moffett et al., 2017).

Here, we investigate the features of the two trophoblast models in more detail to identify which type of trophoblast *in vivo* they most closely resemble. We compare the transcriptomes of these 2D and 3D trophoblast models and validate key markers of *in vivo* trophoblast subtypes at the protein level. Our findings suggest that TSCs closely resemble cells of the column niche, whereas TOs are more similar to VCT. We also determined which products of the six HLA class I genes are expressed by each model. In addition, we found that the 3D model better recapitulates the HLA expression profile of *in vivo* trophoblast and that miRNAs might play a role in HLA class I regulation. Our findings will allow an informed choice of the appropriate *in vitro* model when asking specific biological questions about trophoblast development, differentiation and function.

## RESULTS

### Expression patterns of HLA class I molecules in trophoblast models cultured *in vitro*

**Fig. 1.**
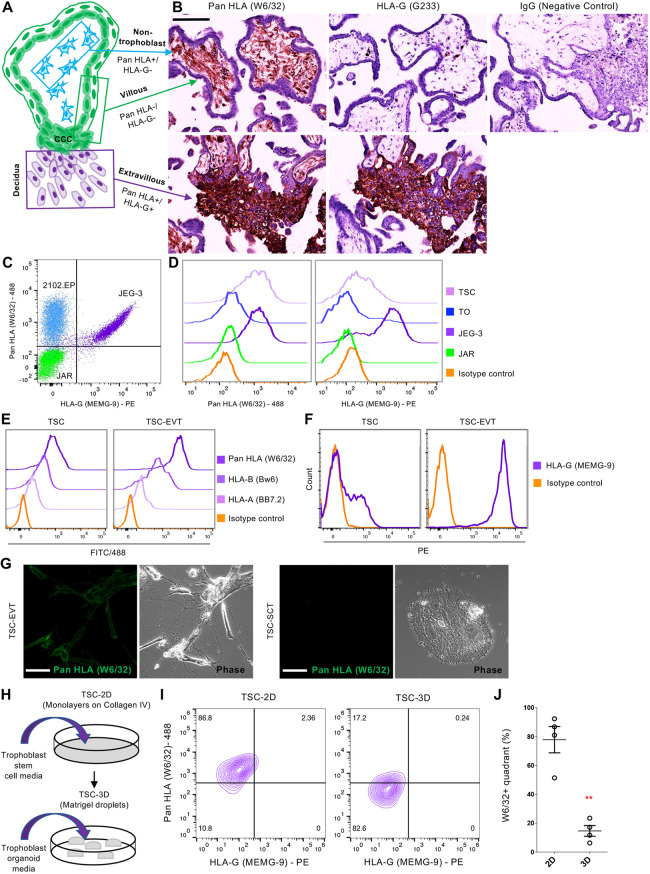
**HLA class I expression in the human placenta and *in vitro* models of human trophoblast.** (A) Illustration of a first-trimester anchoring villus. HLA-null villous cells [SCT, VCT and the base of the cell columns (CCC)] are in green; HLA-C, E and G+ EVT are in purple; and HLA-A+, B+, C+ and HLA-G− nontrophoblast cells of the villous core are in blue. (B) Immunohistochemical staining for HLA class I molecules on acetone-fixed first-trimester placental sections. Here, the pan-class I antibody W6/32 (binds all HLA class I molecules) stains the villous core (nontrophoblast) and the EVT in the cell columns but not VCT or SCT. Staining with G233, specific for HLA-G, increases as cells move away from the villi into the cell column, whereas the entire villus remains negative. (C) HLA profile (using W6/32 and MEMG-9, an HLA-G-specific antibody) of the cell lines used as controls for HLA expression by FACS: JEG-3 (control for HLA profile of extravillous trophoblast); JAR (control for villous trophoblast); and 2102Ep (nontrophoblast control). (D) FACS analysis of W6/32 and MEMG-9 in JAR, JEG-3, TOs and TSCs. TOs have the profile of villous trophoblast (W6/32−/MEMG-9−; *n*=4), whereas TSCs have neither villous nor extravillous profiles [W6/32+/MEMG-9−; *n*=5]. (E) FACS analysis of TSCs grown under proliferative conditions and when differentiated to EVT (*n*=2). Allele-specific antibodies were used to assess HLA-A (BB7.2) and HLA-B (Bw6) expression in a HLA-genotyped TSC line (BTS5). Undifferentiated TSCs express HLA-A and HLA-B, which is maintained after EVT differentiation. (F) FACS analysis demonstrating upregulation of HLA-G in TSCs following EVT differentiation (*n*=3). (G) Live staining for W6/32-Alexa488 on TSCs differentiated to either EVT or SCT (*n*=2). Distinct membrane staining is seen when differentiated to EVT and is absent in SCT. (H) Experimental set-up of the different trophoblast culture conditions. (I) FACS analysis of TSCs grown in 2D versus 3D (passaged more than six times in 3D, *n*=4) with W6/32 and MEMG-9. The number of cells that are W6/32+/MEMG-9− significantly decreases when cultured in 3D. (J) Quantification of the percentage of cells in the W6/32+/MEMG-9− quadrant under 2D or 3D conditions (data are mean±s.e.m., paired two-tailed Student's *t*-test, **P=0.0019). Scale bars: 50 µm in G; 150 µm in B.

We first investigated the expression of HLA class I molecules by the trophoblast models because this is a defining feature of human trophoblast (Faulk and Temple, 1976; King et al., 2000), with a clear difference between VCT, SCT and EVT (Fig. 1A) (Apps et al., 2009; Lee et al., 2016). This was shown using serial sections of first-trimester human placenta stained with a monoclonal antibody (mAb), W6/32 (Brodsky et al., 1979), which recognizes all HLA class I molecules, and G233, an HLA-G-specific mAb (Loke et al., 1997) (Fig. 1B). EVT in the cell columns stained for both W6/32 and G233, confirming HLA-G expression, whereas VCT and SCT did not stain with either mAb. All cells in the mesenchymal villous core stained positively with W6/32 but not with G233, indicating that any HLA class I molecule other than HLA-G could be expressed.

HLA class I genes are polymorphic and have common shared epitopes; it is therefore difficult to work out exactly which allotypes are expressed. By using W6/32, we were able to define whether the trophoblast sample resembles VCT (HLA-null). If there was expression with W6/32, it was then necessary to use locus-specific antibodies for HLA-G, -E and -C (EVT profile) to determine which of these HLA class I genes are expressed. We also tissue typed the individual samples to determine which HLA-A or HLA-B alleles are present and then used HLA-A, -B allele/group-specific antibodies to determine whether they are expressed (Table S1). For all flow cytometric experiments, JEG-3 and JAR choriocarcinoma cells were used as controls for HLA expression for EVT and VCT, respectively (Apps et al., 2009; Sheridan et al., 2020). The embryonal carcinoma cell line, 2102Ep, or primary decidual stromal cells were used as a positive control for HLA-A, -B and -C expression and also served as a negative control for HLA-G (Lee et al., 2016). The specificity of the HLA mAbs used are summarized in Table 1. All control cell lines, and their respective HLA expression patterns, are summarized in Fig. 1C and Table 2. TO, a model for VCT and SCT, were completely HLA null and only expressed HLA-C and HLA-G upon differentiation to EVT (Fig. 1D; Fig. S1A) (Turco et al., 2018; Sheridan et al., 2020). Furthermore, TO remained HLA-B negative when differentiated to EVT (Fig. S1B). In contrast, TSCs (Okae et al., 2018) displayed a pattern of HLA expression that was not characteristic of trophoblast *in vivo* because the former stained positively for the pan-HLA class I marker W6/32, with only a few cells staining for MEMG-9 (specific for HLA-G) (Fig. 1D). This HLA pattern was only observed in nontrophoblast cells of the human placenta *in vivo* (Fig. 1A,B). In addition, HLA expression in TSCs appeared either low/negative or positive depending on the gating strategy used (Fig. S1C,D).

**Table 1.**
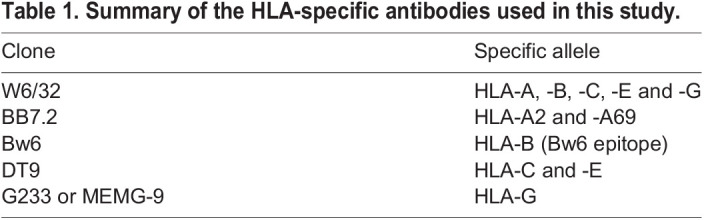
Summary of the HLA-specific antibodies used in this study.

**Table 2.**
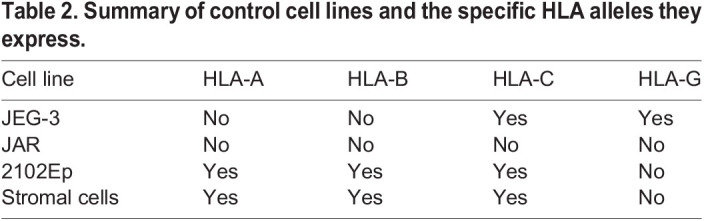
Summary of control cell lines and the specific HLA alleles they express.

### Comprehensive characterization of HLA class I expression in TSCs

To investigate further the unusual expression of HLA class I molecules in TSCs (which display all other defining characteristics of normal trophoblast), we next asked which allotypes of HLA class I genes were expressed. HLA-A, -B, -C and/or -E allotypes could be expressed by TSCs because they are W6/32+, MEMG-9−. The HLA-A and HLA-B alleles present in the TSC lines were determined by HLA DNA genotyping (Table 3; Table S1). Four out of the five TSC lines tested carried an HLA-A*02 allele, and all five had HLA-B alleles encoding the Bw6 epitope. Specific antibodies for HLA-A2 (BB7.2) and HLA-B (Bw6) and an antibody specific for all HLA-C and -E allotypes (DT9) were chosen for analysis by flow cytometry. JEG-3 cells were used as a positive control for pan-HLA class I (W6/32), HLA-C and -E (DT9) and HLA-G (MEMG-9) expression. We found that TSCs expressed HLA-A, -B and -C, but not -G (Fig. S1E). Out of the TSC lines tested, most exhibited HLA-A (three out of four) and HLA-B expression (four out of five) (Fig. 1E; Fig. S1E). One TSC line (CT30) displayed lower expression of W6/32 and was the only line that showed no indication of HLA-A or -B expression (Table 3). Once the TSCs differentiated to EVT (TSC-EVT), HLA-G expression increased to levels similar to those in JEG-3 cells (Fig. 1F), although the TSC-EVT still retained expression of HLA-A and HLA-B (Fig. 1E). Interestingly, when the TSCs differentiated to SCT, HLA expression was downregulated, as shown by live cell staining with W6/32-488 (Fig. 1G). Thus, altering the culture conditions to induce SCT or EVT differentiation affected HLA expression.

**Table 3.**
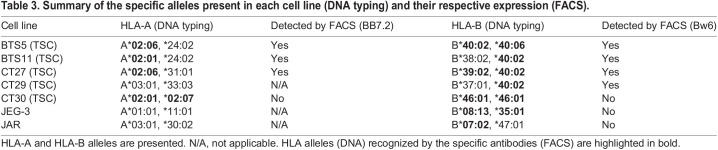
Summary of the specific alleles present in each cell line (DNA typing) and their respective expression (FACS).

We next cultured TSCs using conditions for TOs as an additional comparison (Fig. 1H; Fig. S2A). Our reasoning was that, because TOs fully recapitulate the *in vivo* HLA expression profile, culturing TSCs in 3D might alter their HLA expression. The expression of W6/32 decreased significantly when TSCs were cultured in 3D compared with 2D (Fig. 1I,J; Fig. S2B). This same decrease in W6/32 expression occurred when TSCs were cultured in 3D in their original TSC media, although the cultures could not be passaged long term (Fig. S2B). To test the reverse conditions, TOs were transferred into TSC medium or into 2D culture conditions; however, they were unable to survive long term in either media (TSC or TO) or in 3D in TSC medium (Fig. S2C). Following single cell dispersal, TSC-3D generated more organoids/per cell compared with TO, although individual organoid sizes were similar (Fig. S2D).

### Transcriptome profile of trophoblast models *in vitro*

**Fig. 2.**
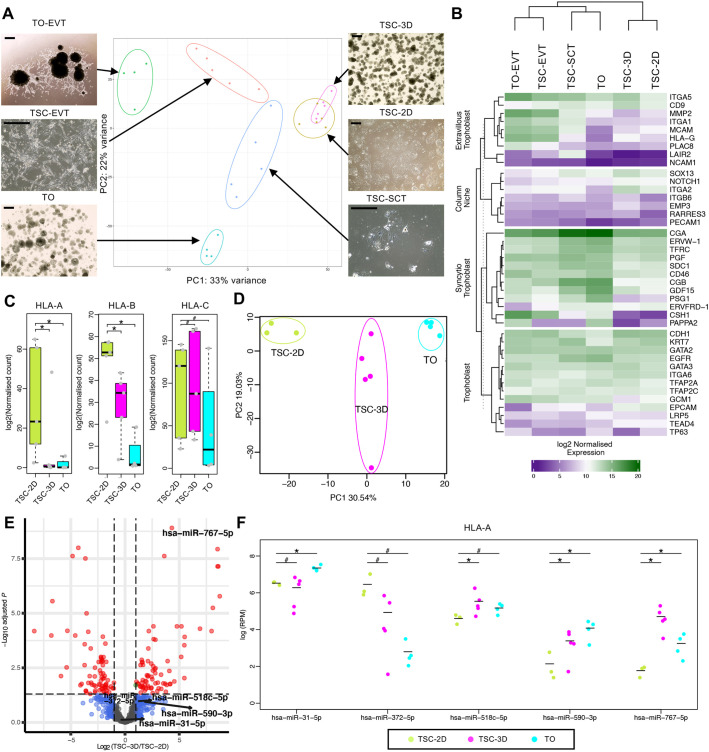
**Transcriptomic and miRNA profiles of the human trophoblast models.** (A) PCA (with the top-2000 most-variable genes) of the trophoblast cell models: TOs (*n*=4), TO-EVT (*n*=4), TSC-2D (*n*=5), TSC-3D (*n*=5), TSC-EVT (*n*=5) and TSC-SCT (*n*=5). A representative image from each sample type is shown. (B) A heatmap of specific markers for each trophoblast subtype from the human first-trimester placenta. The markers are divided into the following groups: pan-trophoblast, SCT, cell column niche and EVT. Hierarchical clustering is shown based on log2 normalized expression. (C) Expression of classical HLA class I genes in TSC-2D, TSC-3D and TOs. Expression levels of *HLA-A* and *HLA-B* are significantly higher in TSC-2D compared with TSC-3D (*HLA-A P*=0.028; *HLA-B P*=0.048) and TOs (*HLA-A P*=0.018; *HLA-B P*=0.008). No significant differences were seen for transcripts of *HLA-C*. Data are mean±s.e.m. (D) PCA of small RNA (miRNA) profiles between TSC-2D (*n*=3), TSC-3D (*n*=5) and TOs (*n*=4). (E) Volcano plot of differentially expressed miRNAs between TSC-2D and TSC-3D. miRNAs that are targets of *HLA-A* are highlighted (five in total). (F) Expression levels of the five specific miRNAs that target *HLA-A* in TSC-2D, TSC-3D and TOs. Significance in C and F estimated by one-sided Wilcoxon test (*P<0.05; ^#^*P*>0.05). Scale bars: 500 µm in A.

Next, to analyze differences in addition to their HLA expression patterns, we compared the transcriptomes of TSCs and TOs. We performed RNA sequencing (RNA-seq) on six different groups: (1) TOs (*n*=4 donors); (2) TOs differentiated to EVT (TO-EVT, *n*=4 donors); (3) TSCs (TSC-2D, *n*=5 donors); (4) TSCs cultured in 3D in TO medium (TSC-3D, *n*=5 donors); (5) TSCs differentiated to EVT (TSC-EVT, *n*=5 donors); and (6) TSCs differentiated to SCT (TSC-SCT, *n*=5 donors). All six groups showed different morphologies in culture (Fig. 2A). A principal component analysis (PCA) was completed using the top-2000 most-variable genes (Fig. 2A). As expected, TOs, which comprise VCT and SCT, clustered closest to TSC-SCT. Similarly, TO-EVT clustered most closely with TSC-EVT. TSCs differentiated to EVT and SCT were not as uniformly differentiated (specifically one patient line, CT27, displayed similar expression and showed mixed morphology when differentiated to EVT and SCT) as the respective TO groups. We also found that TSC-3D clustered almost identically to TSC-2D despite the different culture conditions.

We subsequently generated heatmaps using the following criteria: (1) known markers specific for trophoblast subtypes found *in vivo* (Damsky et al., 1992; Apps et al., 2011; Haider et al., 2016; Lee et al., 2016, 2018; Chang et al., 2018; Turco et al., 2018; Vento-Tormo et al., 2018; Okae et al., 2018; Knöfler et al., 2019; Turco and Moffett, 2019; Meinhardt et al., 2020) (Fig. 2B); (2) a complex heatmap by using a one-against-all DEseq2 analysis (Fig. S3; Tables S2-S7); or (3) the top-250 most-differentially expressed genes (DEGs) between samples (Fig. S4A). Log_2_ normalized expression values were used for all heatmaps. Hierarchical clustering based upon trophoblast markers indicated a similar pairing as found in the PCA (Fig. 2B). TO-EVT and TSC-EVT were most similar to each other and showed high expression of the majority of pan-trophoblast and EVT markers. Of note, expression of markers of SCT (*CGA*, *PGF* and *CSH1*) were also high in both TSC-EVT and TO-EVT. TO-EVT did still contain some syncytium, and some off-target differentiation in TSC-EVT was likely. TOs and TSC-SCT were closely aligned and showed the highest expression of SCT markers. TSC-2D and TSC-3D clustered closely and resembled trophoblast and the niche in the cell columns. Interestingly, when only using the top-250 DEGs, the hierarchical clustering indicated that organoids were most like each other, and TSCs all clustered separately (Fig. S4A). Transcripts for *HLA-A* and *HLA-B* were significantly reduced in TSC-3D (*HLA-A P*=0.028 and *HLA-B P*=0.048) and TOs (*HLA-A P*=0.018 and *HLA-B P*=0.008) compared with TSC-2D (Fig. 2C) in line with protein expression levels. Transcripts for *HLA-C* did not differ between groups and displayed increased heterogeneity between different patient-derived lines (Fig. 2C), which could be associated with the increased turnover rate of *HLA-C* (McCutcheon et al., 1995). Genes commonly associated with MHC class I regulation include: (1) transcription factors (*NLRC5*, *RFX5*, *RFXAP* and *RFXANK*); and (2) those involved in binding, transport and peptide loading (*TAPBP*, *PDIA3*, *CALR*, *TAP1* and *TAP2*) (Fig. S4B) (Jongsma et al., 2019). Transcript levels of *NLRC5*, the master regulator of HLA class I genes, were significantly higher in TOs compared with TSC-2D and TSC-3D, indicating that mRNA expression did not correlate with the functional expression levels (protein expression in Fig. 1 and Fig. S1) (Neerincx et al., 2012). *CIITA*, the master regulator of MHC class II in antigen-presenting cells, was not expressed.

### Small RNA (miRNA) profile of trophoblast models *in vitro*

Given the similarities in transcriptomes between TSC-2D and TSC-3D, we next asked whether there were any differences in miRNAs that could explain the differing HLA expression levels. Small RNA-seq was analyzed comparing TSC-2D, TSC-3D and TOs. Unfortunately, two of the five TSC-2D samples were technical outliers, with either poor enrichment of miRNAs or contamination with larger RNA species. These samples were removed prior to downstream analysis. A PCA indicated that all groups clustered separately (Fig. 2D). We found 99 differentially expressed miRNAs between TSC-2D and TSC-3D [Benjamini and Hochberg (BH) corrected *P*<0.05, fold change >2] (Fig. 2E). Given that transcripts of *HLA-A* showed the strongest downregulation, we investigated which miRNAs were expected to target *HLA-A* transcripts (Wong and Wang, 2015). These predicted miRNAs are highlighted on the volcano plot (Fig. 2E). Three of the five predicted miRNAs (hsa-miR-518c-5p, hsa-miR-590-3p and hsa-miR-767-5p) were increased in TSC-3D samples compared with TSC-2D (Fig. 2F). This trend was conserved in both 3D models (i.e. TSC-3D and TOs) compared with 2D. This highlights a potential mechanism that is enhanced in 3D cultures to target and suppress expression of *HLA-A* transcripts.

### Morphological and functional characterization of TSC-3D and TOs

**Fig. 3.**
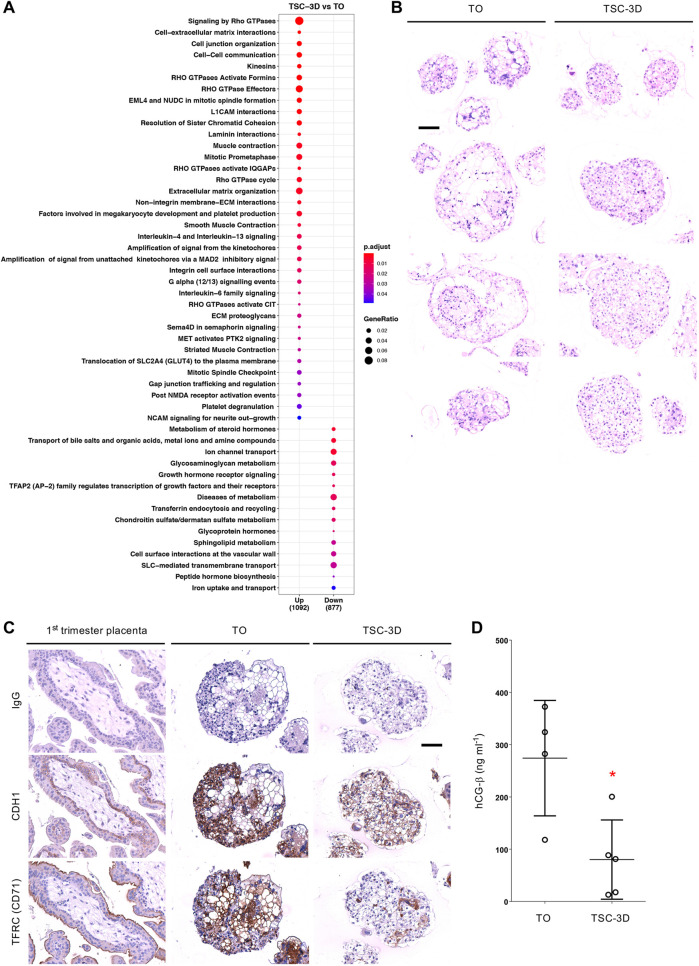
**Morphological and functional characterization of TSC-3D and TOs.** (A) Enriched pathway analysis demonstrates upregulated and downregulated pathways in TSC-3D compared with TOs. The gene ratio is the number of DEGs identified divided by the total genes in each pathway. Blue and red colors signify the adjusted *P*-values. (B) H&E of TOs (*n*=4) and TSC-3D (*n*=5) of different sizes. (C) IHC of CDH1 (mononuclear trophoblast marker) and TFRC (SCT marker). For reference, first-trimester villi are shown stained for each marker. CDH1 is localized to VCT and TFRC to the syncytial brush border. TOs (*n*=2) are positive for both markers, with the outer cells stained for CDH1 and the inner cells lining the luminal spaces stained for TFRC. In contrast, the majority of the cells in TSC-3D (*n*=2) are positive for CDH1, with only small foci positive for TFRC. (D) ELISA to measure the production by SCT of the pregnancy hormone, hCG-β, by TOs (*n*=4) and TSC-3D cultures (*n*=5) (48 h conditioned medium collected from cultures with comparable numbers of organoids at similar sizes). TSC-3D secrete significantly less hCG-β compared with TOs (data are mean±s.e.m., unpaired two-tailed Student's *t*-test, *P=0.029). Scale bars: 100 µm in B,C.

The transcriptome data suggested that, even when cultured in identical conditions, TO and TSC-3D have different identities, further demonstrated with an enriched pathway analysis (Fig. 3A; Table S8). Many of the biological processes upregulated in TSC-3D were related to extracellular matrix remodeling and cell-cell interactions (Fig. 3A; Table S8). Conversely, terms upregulated in TOs were related to transport, metabolism and hormone signaling (Fig. 3A; Table S8). A PCA using the top-2000 most-variable genes between TOs and TSC-3D revealed that multiple SCT markers (*PAPPA2*, *PSG1*, *VGLL3*, *PAPPA*, *PSG3*, *PSG8*, *PLAC4* and *CGB5*) accounted for the separation between the two groups (Fig. S5A) and were significantly upregulated in TOs (Table S9). To further address whether TSC-3D can form SCT, as TOs do, we stained cultures with Hematoxylin and Eosin (H&E) (Fig. 3B), performed immunohistochemistry (IHC) for CDH1 and TFRC (Fig. 3C; Fig. S5B) and tested culture supernatants with a human chorionic gonadotropin (hCG-β) ELISA (Fig. 3D). These experiments showed that SCT formation in TSC-3D was significantly lower than in TOs. Thus, TOs showed more-vigorous spontaneous syncytial formation compared with TSCs when grown under the same conditions.

**Fig. 4.**
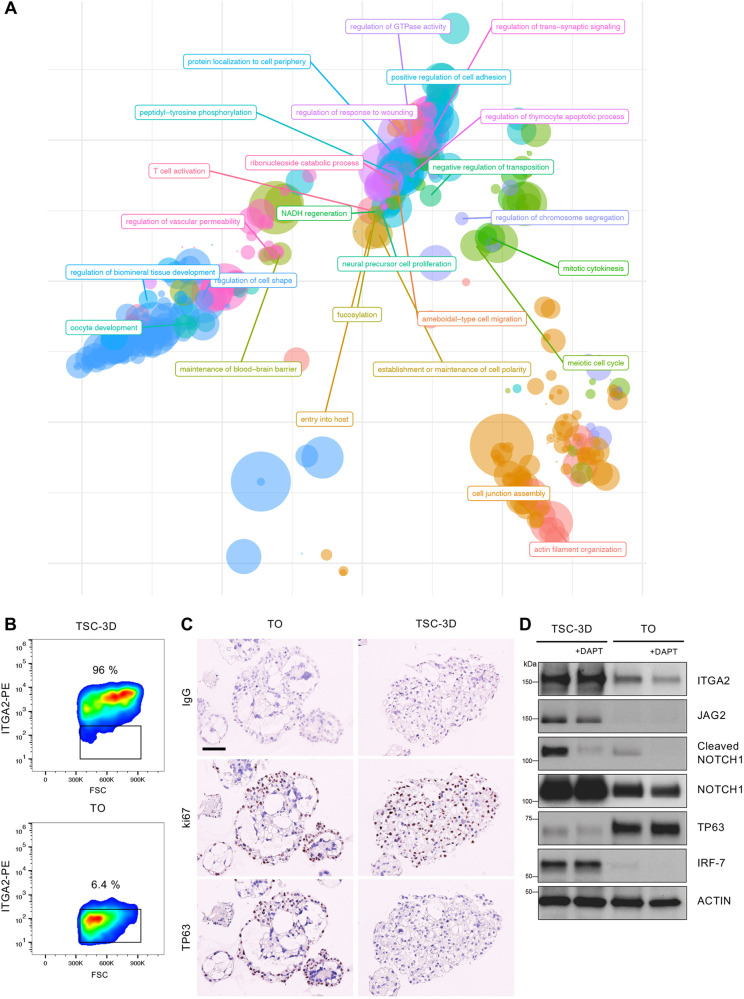
**Characterization of markers specific for cell columns in TSC-3D and TOs.** (A) GO semantic analysis for enriched biological pathways upregulated in TSC-3D compared with TOs. (B) FACS analysis for ITGA2 in TSC-3D and TOs. The gate is drawn on the isotype control (*n*=3). (C) IHC for a proliferation marker (Ki67) and a villous trophoblast marker (TP63) in TOs (*n*=2) and TSC-3D (*n*=2). Both groups contain Ki67+ cells. TP63+ cells are present in TOs but absent from TSC-3D. (D) TSC-3D and TOs were cultured in the presence or absence of a NOTCH signaling inhibitor (DAPT, 20 µM) for 8 days (*n*=2). Western blots indicated differences in active NOTCH signaling (cleaved NOTCH1), and JAG2 (NOTCH ligand) between TSC-3D and TOs. DAPT treatment inhibited active NOTCH signaling in both models. ITGA2 and IRF7 are expressed at high levels in TSC-3D, whereas TP63 is upregulated in TOs. Actin is the loading control. Scale bar: 100 µm in C.

A gene ontology (GO) semantic analysis was performed to visualize the functional similarities of the enriched pathways upregulated in TSC-3D compared with TOs (Fig. 4A, Table S10). Terms related to cell shape, cell adhesion, polarity and development were highly represented. Downregulated terms (i.e. upregulated in TOs) are detailed in Table S11. Again, common parent terms were related to metabolic, catabolic and transport processes. Next, we asked whether these TSC-3D more closely recapitulate the column niche rather than villous trophoblast. *In vivo*, ITGA2 and NOTCH1 are characteristically expressed in the column niche (Haider et al., 2014, 2016; Lee et al., 2018). Flow cytometric analysis confirmed that TSC-3D contained a higher percentage of ITGA2+ cells compared with TOs (Fig. 4B). High ITGA2 expression has also been demonstrated in TSCs cultured in 2D (Cinkornpumin et al., 2020). The forward scatter (FSC) demonstrated that the cells isolated from TSC-3D were larger than those from TOs. Another defining feature of the cytotrophoblast cell columns is the shift from TP63+ villous trophoblast to TP63− proliferating cells (Haider et al., 2016). We performed IHC on serial sections for Ki67 (to determine general proliferation) and TP63 (to mark proliferating villous trophoblast). TOs contained Ki67+/TP63+ cells, whereas TSC-3D only contained Ki67+ cells (Fig. 4C; Fig. S5C). Previously, NOTCH1 was shown to reduce TP63 expression through the downregulation of IRF6 or IRF7 (Nguyen et al., 2006; Haider et al., 2016). To investigate this further, we treated TSC-3D and TOs with a small-molecule inhibitor of NOTCH1 (DAPT). Western blotting confirmed that there is a distinct switch in protein expression of ITGA2 and TP63 between TSC-3D and TOs (Fig. 4D). Furthermore, TSC-3D exhibited higher levels of active NOTCH1 signaling, demonstrated by expression of cleaved (active) NOTCH1 and the presence of JAG2 (a NOTCH1 ligand). TSC-3D also expressed high levels of IRF7. As anticipated, DAPT treatment reduced active NOTCH1 signaling in both TSC-3D and TOs, but did not affect levels of any other protein. Unexpectedly, the reduction in NOTCH1 signaling did not alter either IRF7 or TP63 expression in TSC-3D. Together, these results indicate that TSC-3D most closely resemble the column niche.

## DISCUSSION

Here, we compared different *in vitro* models of human first-trimester trophoblast so that informed choices can be made when posing biological questions. We first characterized the HLA profiles of the two trophoblast models derived from primary tissue, because these have been invaluable in categorizing cellular identity from cells at the maternal-fetal interface. The different HLA expression in the choriocarcinoma cell lines, JEG-3 or BeWO, compared with JAR was the first hint that villous trophoblast is HLA null, whereas EVT expresses HLA class I molecules (Trowsdale et al., 1980). More recently, expression of classical HLA-A and -B antigens by cells induced from BMP-stimulated embryonic stem cells raised questions about whether these cells were trophoblast or mesoderm cells (Bernardo et al., 2011; Roberts et al., 2014). We found that there was also expression of classical HLA molecules in TSCs, in contrast to the initial study, which reported low or negligible expression. The discrepancy in the results is possibly because of the gating strategies used (Okae et al., 2018). Stromal cells were used in the initial report as a positive control. When we repeated this experiment, using the same gating strategy and stromal cells as our HLA-positive control, we also found that HLA expression in TSCs appeared to be low (Fig. S1C,D). However, by drawing a gate on two negative controls (isotype control and HLA-negative JAR cells) and one HLA-positive control (JEG-3 cells), TSCs did express HLA class I molecules. This highlights the importance of choosing appropriate controls for HLA analysis, particularly because trophoblast displays a unique HLA profile. Another group also reported expression of HLA class I molecules (determined by W6/32) in TSCs (Cinkornpumin et al., 2020).

Many groups have also established that TSCs can be derived from naïve or extended potential pluripotent stem cells (Castel et al., 2020; Cinkornpumin et al., 2020; Dong et al., 2020; Liu et al., 2020; Guo et al., 2021; Io et al., 2021). Each report concludes that EVT differentiated TSCs form HLA-G-positive cells, yet full HLA profiles have been missing from these reports. In agreement with our findings, TSCs derived from conversion of naïve pluripotent stem cells are also reported to express W6/32 (Cinkornpumin et al., 2020; Liu et al., 2020). Only one of these reports indicated that TSC-derived cells were completely negative for W6/32 (Io et al., 2021) and others did not assess W6/32 expression (Castel et al., 2020; Dong et al., 2020; Guo et al., 2021). Variability in specific methods used by each group might also contribute to the different HLA Class I expression levels found.

We also revealed a further complexity pointing to a role for mechanical regulation of HLA expression that might explain some of the previous findings. TOs remained HLA null unless stimulated to differentiate to EVT. In contrast, TSCs (which are HLA null *in vivo*) upregulated expression when cultured in 2D and downregulated expression in 3D. However, we did not find a complete absence of HLA expression in TSCs in any situation. It is known that 2D culture conditions do not always recapitulate, or maintain, all features of their *in vivo* equivalents as well as 3D culture systems (Schutgens and Clevers, 2020). Others have also demonstrated the importance of tissue/mechanical stiffness in regulating miRNAs that target cytoskeletal, adhesive and extracellular matrix proteins (Moro et al., 2019). Therefore, we reason that mechanical cues (Wagh et al., 2021) affect HLA transcription and expression. In particular, miRNAs that were predicted targets of *HLA-A* were upregulated under 3D conditions compared with 2D and might be involved in regulating HLA. In support of this hypothesis, of the miRNAs that were differentially expressed between TSC-2D and TSC-3D, hsa-miR-518c-5p was shown by another group to be more highly expressed in samples with lower HLA expression (Io et al., 2021). HLA-G expression can be targeted by miRNAs in JEG-3 cells, as well as HLA-ABC expression in nontrophoblast cells (Gao et al., 2013; Friedrich et al., 2020). In addition, it is unlikely that trophoblast HLA transcription is regulated in the same manner as nontrophoblast cells because of their unique HLA profile (HLA-C was upregulated with differentiation to EVT, whereas HLA-A and HLA-B remained silenced). Our findings on the importance of a 3D microenvironment and its relation to HLA expression could also be applied to other 2D trophoblast models (Lee et al., 2016). Pluripotent stem cells treated with BMP4 and signaling inhibitors of activin and FGF2 are widely used as a model for trophoblast (Amita et al., 2013; Horii et al., 2016). Although they recapitulate many features of normal trophoblast, their HLA expression pattern is also different. However, the molecular mechanisms underpinning these findings need further study.

We looked at other characteristics to compare these models with the corresponding phenotype and location of trophoblast *in vivo*. Although both models contain proliferative trophoblast *in vitro* (Haider et al., 2018; Okae et al., 2018; Turco et al., 2018), the exact location and identity of human trophoblast progenitor cells (or stem cells) *in vivo* remains unknown. Although the models are both bipotent, capable of generating EVT and SCT, there may be a bias in directionality. Spatially, progenitors in the column niche seem likely to favor differentiation into EVT, whereas those in the villi (VCT) would fuse into SCT. TSCs, with high expression of EPCAM and ITGA2, closely recapitulated the column niche (Cinkornpumin et al., 2020). When cultured in 3D, they maintained a higher proportion of ITGA2+ cells and formed significantly less SCT compared with TOs (Figs 3 and 4). Other features of TSC-3D point to their resemblance to the column niche: namely, active NOTCH1 signaling, TP63 downregulation and enrichment of GO terms related to adhesion, invasion and migration. However, inhibition of NOTCH1 did not rescue TP63 expression. It is possible that short-term (<10 days) NOTCH1 inhibition was not sufficient to abrogate IRF7 and consequently rescue TP63 expression. Alternatively, there might be other pathways upregulating IRF7 that also need to be suppressed. In contrast, TOs appeared enriched for VCT progenitors and spontaneously generated abundant SCT. Recently, the formation of SCT from different types of TSC grown in 3D was reported, but these were not compared to TOs (Castel et al., 2020; Saha et al., 2020; Io et al., 2021). Thus, if models of SCT are required, careful choice of the *in vitro* trophoblast model is needed.

In conclusion, the two models of first-trimester trophoblast differed in their expression of HLA and other genes. Our findings will help researchers choose the most-appropriate model to study trophoblast development, function and response to insults. The exact pathways controlling TP63 expression and the molecular switches vital for VCT-to-cell column conversion could be unraveled using TSCs because they closely resemble the column niche (Fig. 4). With their abundant SCT formation, TOs are more appropriate for studies of pathogens that enter the villi from maternal blood in the intervillous space to infect the fetus *in utero*, and studies involving drug metabolism or hormone secretion (Fig. 3). The most appropriate model to investigate interactions of EVT with immune cells, particularly uterine NK cells and macrophages, are TOs because they differentiate to HLA-G+ HLA-C+ EVT. TSCs never completely lost expression of classical HLA-A and HLA-B molecules even upon EVT differentiation (Fig. 1). However, the complexity of TOs means that it is harder to dissect a response by one specific cell type. Lineage-specific effects could be strategically assessed in the TSC model because TSC, EVT and SCT lineages can be studied in parallel. From a cost and time perspective, TSCs are much easier to scale-up and biobank (in 2D and 3D), whereas TOs take much longer to grow and expand in culture (Fig. S2D). Being aware of all of the advantages and limitations of these newly derived models will greatly enhance the field of trophoblast research and lead to a better *in vitro* representation of the *in vivo* situation.

## MATERIALS AND METHODS

### Patient samples

All tissue samples used for this study were obtained with written informed consent from all participants in accordance with the guidelines in The Declaration of Helsinki 2000. Tissue samples used for our study were obtained from Addenbrooke's Hospital (6-11 weeks gestation) under ethical approval from the Cambridge Local Research Ethics Committee (04/Q0108/23), which is now incorporated into the overarching ethics permission given to the Centre for Trophoblast Research biobank for the ‘Biology of the Human Uterus in Pregnancy and Disease Tissue Bank’ at the University of Cambridge under ethical approval from the East of England–Cambridge Central Research Ethics Committee (17/EE/0151).

### Derivation and differentiation of trophoblast organoids from human placental tissue

In total, seven different patient-derived lines were used in this study. We have previously published a detailed protocol of the derivation, maintenance and differentiation of human TO cultures (Sheridan et al., 2020). For transcriptome analysis, TO and TO-EVT cultures were collected in parallel when TO-EVT showed outgrowths in >50% of the organoids (typically between 7 and 10 days of differentiation). Briefly, TO and TO-EVT cultures were removed from Matrigel (Corning) with Cell Recovery Solution (Corning, 354253) and then washed and resuspended into QIAzol lysis reagent (Qiagen). When TO-EVT samples were collected, they contained both the EVT outgrowths and all the remaining organoids. Short-term 2D conversion of TOs was achieved by plating a single cell suspension onto a Matrigel-coated (0.1 mg/ml) six-well culture plate. Cell monolayers were cultured in trophoblast organoid medium (TOM) [Advanced DMEM/F12 (Thermo Fisher Scientific), N2 supplement (Thermo Fisher Scientific, 17502048; at manufacturer's recommended concentration), B27 supplement minus vitamin A (Thermo Fisher Scientific, 12587010; at manufacturer's recommended concentration), Primocin 100 μg/ml (InvivoGen, ant-pm), *N*-acetyl-L cysteine 1.25 mM (Sigma-Aldrich, A9165), L-glutamine 2 mM (Sigma-Aldrich, 25030024), recombinant human EGF 50 ng/ml (Peprotech, AF10015), CHIR99021 1.5 μM (Tocris, 4423), recombinant human R-spondin-1 80 ng/ml (R&D systems, 4645-RS-01M/CF), recombinant human FGF-2 100 ng/ml (Peprotech, 100-18B), recombinant human HGF 50 ng/ml (Peprotech, 100-39), A83-01 500 nM (Tocris, 2939), prostaglandin E2 2.5 μM (Sigma-Aldrich, P0409), and Y-27632 2 μM (Millipore, 688000)].

### TSC culture and differentiation

Five different patient-derived TSC lines were used in this study (BTS5, BTS11, CT27, CT29 and CT30) and were cultured according to the previously published protocol (Okae et al., 2018) with the following modifications. The concentration of CHIR99021 was doubled to 4 µM to help prevent spontaneous differentiation to HLA-G+ EVT. For TSC-SCT differentiation, the collagen IV concentration for plate-coating was increased to 5 µg/ml. TSC-SCT samples were collected at day 6 and TSC-EVT were collected at day 8 for downstream analysis. Conversion of TSCs to 3D cultures was achieved by passaging a single cell suspension, plating into 25 µl droplets of Matrigel and overlaying with TOM media (detailed above). TSC-3D were then cultured in the same manner as the TOs (Sheridan et al., 2020). TSC-3D were passaged every 7-10 days and then collected for RNA extraction in the same manner as TOs.

### Isolation of stromal cells

Samples of decidual tissue were used for the isolation of decidual stromal cells (Male et al., 2012). Cells were used for fluorescence activated cell sorting (FACS) experiments after passage 2. Stromal cells were cultured in Advanced DMEM/F-12 supplemented with 10% (v/v) fetal bovine serum (FBS), 2 mM l^−1^ glutamine, 10 units ml^−1^ penicillin, 100 µg ml^−1^ streptomycin and 2 mg ml^−1^ gentamicin (all Thermo Fisher Scientific). Culture medium was replaced every 2-3 days. Approximately 4-6 days after plating, cells were removed from tissue culture flasks with TrypLE (Gibco) to be either passaged at a ratio of 1:3 or used as a control for flow cytometry experiments.

### Cell lines

Choriocarcinoma cell lines, JEG-3 and JAR, and embryonal carcinoma line 2102Ep (used only as control cell lines for experiments in this study) were purchased from the American Type Culture Collection (ATCC) in 2015. Cells were expanded and frozen stocks were immediately made within a few passages of receipt of these cell lines. Early passage numbers were thawed for this study. Cells were cultured in RMPI-1640 (Thermo Fisher Scientific) supplemented with 10% (v/v) FBS, 2 mM l^−1^ glutamine, 10 units ml^−1^ penicillin, 100 µg ml^−1^ streptomycin and 2 mg ml^−1^ gentamicin. Culture medium was replaced every 2-3 days. Approximately 4-6 days after plating, cells were removed from tissue culture flasks with TrypLE to be either passaged at a ratio of 1:3 or used as a control for flow cytometry experiments.

### Immunohistochemistry

Organoids were formalin fixed and embedded as previously described (Turco et al., 2017). Immunohistochemistry on sections of TOs and TSC-3D was performed using heat-induced epitope retrieval buffers (Menarini) and Vectastain avidin-biotin-HRP reagents (Vector Lab, PK-6100) as previously described (Turco et al., 2017). Primary antibodies (Table S12) were replaced with equivalent concentrations of isotype-matched mouse or rabbit IgG for controls. For HLA staining of first-trimester placenta, frozen placental sections were fixed in acetone prior to staining. Images were captured with an EVOS M5000 Imaging System (Thermo Fisher Scientific).

### Live staining and confocal microscopy

TSCs were plated on 35-mm ibidi μ-dishes and differentiated to either EVT or SCT. Once differentiation was complete, the cells were incubated with W6/32-488 conjugated antibody (Biolegend, 311413; 1:150 diluted in DMEM:F12) for 30 min at 37°C. Cells were washed twice with PBS and culture media was replaced before imaging. Images were captured with a Zeiss LSM 700 Confocal Laser Scanning Microscope with ZEN Microscope Software.

### ELISA

For ELISA, 48 h conditioned media was collected from TO and TSC-3D cultures and stored at −20°C until use. hCG-β ELISA (Abcam, ab108638) was performed following the manufacturer's instructions. Samples were run in duplicate alongside nonconditioned media controls and hCG-β standards. The concentration of hCG-β was calculated from the line formula of the standard plots in Microsoft Office Excel.

### Flow cytometry

Single cell suspensions of TOs and TO-EVT were prepared as previously described (Sheridan et al., 2020). TSC-3D cultures were removed from Matrigel with Cell Recovery Solution and sequentially dissociated with TrypLE at 37°C for 5 min intervals. 2D cultures were directly dissociated with TrypLE at 37°C for 3-8 min. Cells were washed in medium containing FBS and passed through a Falcon 40-μm cell strainer. Cells were blocked with human IgG (Sigma-Aldrich, I4506) in Dulbecco's PBS (Thermo Fisher Scientific, 14190136) with 1% FBS before labeling with specific antibodies or isotype-matched controls (Table S12). LIVE/DEAD Fixable Far Red Dead Cell Stain (Life Technologies, L10119) was used for live/dead discrimination. Data were acquired using the Cytek Development DxP8 (488/637/561) or the Attune NxT. Data were analyzed in FlowJo (Tree Star) and all compensation was applied digitally after acquisition.

### Western blot

Total cell extracts were prepared in radioimmunoprecipitation assay buffer (20 mM Tris-HCL, pH 8.0, 137 mM NaCl, 1 mM MgCl2, 1 mM CaCl2, 10% glycerol, 1% NP-40, 0.5% sodium deoxycholate, 0.1% sodium dodecyl sulfate), containing a protease inhibitor cocktail (Sigma-Aldrich, P2714), and incubated at 4°C for 1 h. Western blotting was performed as previously described (Perez-Garcia et al., 2021). In brief, protein lysates were resolved by sodium dodecyl sulfate polyacrylamide gel electrophoresis (SDS-PAGE) and transferred onto polyvinylidene difluoride membrane (Immobilon-P, Millipore). Membranes were blocked with 5% milk powder and incubated with primary antibody overnight at 4°C, followed by horseradish peroxidise (HRP)-conjugated secondary antibodies. Blots were probed with the following antibodies: anti-ITGA2 (1:1000, Abcam, ab181548), anti-JAG2 (1:1000, Cell Signaling Technology, 2210), anti-Cleaved NOTCH1 (1:1000, Cell Signaling Technology, 4147), anti-NOTCH1 (1:1000, Cell Signaling Technology, 3608), anti-TP63 (1:1000, Abcam, ab124762), anti-IRF7 (1:1000, Cell Signaling Technology, 4920) and anti-β actin (1:5000, Abcam, ab6276). Horseradish peroxidase-conjugated secondary antibodies (used at a dilution of 1:3000) were sourced from Bio-Rad. Detection was carried out with enhanced chemiluminescence reaction (GE Healthcare, RPN2209) using standard X-ray films.

### DNA extraction and quantification

The QIAamp DNA Blood Mini kit (Qiagen, 51104) was used to extract genomic DNA from TOs, TSC lines and JEG-3/JAR cell lines for HLA tissue typing. DNA quality and concentration were determined in a Nanodrop ND-1000 Spectrophotometer.

### HLA typing

The DNA for HLA genotyping was processed via the workflows of a European Federation for Immunogenetics (EFI)-accredited Clinical Histocompatibility Laboratory. Low-resolution typing of the HLA-A, HLA-B and HLA-C genes was achieved with LABType kits (One Lambda), which rely on reaction patterns observed when sequence-specific DNA probes immobilized on fluorescent X-MAP polystyrene beads (Luminex) hybridize to biotin-labeled multiplexed gene-specific PCR amplicons. The hybrids were detected with a Liquichip 200 fluorimeter (Qiagen) and HLA allele assignment was performed using HLA Fusion software (One Lambda). Ultra-high-resolution typing of HLA-A, HLA-B and HLA-C was achieved with an ‘in-house’ third-generation sequencing pipeline using Single Molecule, Real-Time (SMRT) DNA sequencing technology (Pacific Biosciences) as previously described (Turner et al., 2018). Typing results are detailed in Table 3 and Table S1. Details of the World Health Organization (WHO) nomenclature for factors of the HLA system, and Bw6 epitope determination can be found at www.hla.alleles.org.

### RNA extraction, quantification and quality control

Total RNA was isolated using the miRNeasy isolation kit (Qiagen, 217004) with on-column DNase digestion (Qiagen, 79254). RNA quality was assessed on the Agilent 2100 bioanalyzer (Thermo Fisher Scientific). The RNA integrity number (RIN) of each tested sample was greater or equal to 8.

### Illumina TruSeq RNA library preparation, sequencing and analysis

mRNA and small RNA-seq was run on the NextSeq 500 (Illumina) using a 75-cycle high output, which generated approximately 400 million reads per run. Library preparation for bulk mRNA sequencing was performed using the Truseq stranded mRNA library kit (Illumina) following the manufacturer's protocol. Library preparation for small RNA-seq was performed by using the Bioo Nextflex protocol (NEXTFLEX Small RNA-seq Kit v3 for Illumina Platforms). Data were aligned to the GRCh38 human genome (iGenomes, NCBI). The alignment and quality control were processed using the Nextflow (Di Tommaso et al., 2017; Ewels et al., 2020) pipeline (version 20.01.0, https://nf-co.re/rnaseq) with the option ‘--aligner hisat2’. All scripts, with details of software versions, a pipeline usage report and expression count files, are freely available from https://github.com/CTR-BFX/Sheridan_Turco.

Differential gene expression was performed with the DESeq2 (Love et al., 2014) package (v1.26.0, R v3.6.2) (Team, 2020) and read counts were normalized on the estimated size factors with the same package. PCA was performed using the top-2000 most-variable genes using variance stabilizing transformed expression for gene counts. For each contrast, DEGs with BH adjusted *P*-values <0.05 were identified. Significant DEGs from each comparison were selected based on the adjusted *P*-values <0.05; absolute log2FoldChange was greater than 1. For heatmaps, gene-level transcript expression values were derived by log2 normalized transformed values. ComplexHeatmap (v 2.6.2) was applied to generate the heatmap (Gu et al., 2016). To identify the key markers of each sample type (group) and generate unbiased clustering across six groups, one-against-all DESeq2 analysis was performed. Gene lists are reported for each specific group in Tables S2-S7. The top upregulated markers from each comparison were selected based on their order log2FoldChange and adjusted *P*-value.

GO and GO semantic similarity pathway analysis were performed using R package clusterProfiler (version 3.14.3) (Yu et al., 2012), GOsemSim (v 2.16.1) (Yu et al., 2010) and rrvgo (v 1.2.0) (Sayols, 2020). Significant DEGs identified using DESeq2 analysis between TSC-3D and TOs were used as input. In order to check the enriched biological pathways for both up- and downregulated DEGs identified as input, the ‘compareCluster’ function was used with the default clusterProfiler algorithm coupled with Fisher's exact test (*P* ≤0.05, q ≤0.05). Unbiased biological process (BP) GO-enriched pathway analysis was also performed for all up- and downregulated DEGs. Based on the number of BPs identified, GO semantic similarity was performed to cluster genes into different clusters based on their functional similarity, and used to measure the similarities among all, up- and downregulated GO BP terms to reduce redundancy. GO plots were drawn using R package ggplot2 (version 3.3.2) and enrichplot (v 1.10.2).

Normalized read counts were used for the statistical analysis of the mRNA abundance of key genes. Raw sequencing reads are deposited in EMBL-EBI ArrayExpress with experimental code E-MTAB-10429.

### Small RNA (miRNA) analysis

For sequencing reads from TSC-2D, TSC-3D and TO samples, adapters were first removed using Cutadapt v3.3 (Martin, 2011) (adapter sequence was specified as TGGAATTCTCGGGTGCCAAGG), followed by trimming four bases from either end of each read according to the library preparation protocol. Only trimmed reads with the length range of 15-32 nucleotides (nt) were kept for downstream analyses. Before mapping, sequencing reads with identical sequences from each sample were collapsed into one read, with the copy number recorded. The collapsed reads were then mapped to known miRNA precursors from miRBase release 22 (Kozomara et al., 2019) using Bowtie v1.3.0 (parameters: -v 1 -a -m 5 --best --strata --norc) (Langmead et al., 2009). The reads with at least two copies and aligned within three nt of the annotated 5′-position of the mature miRNAs were retained for miRNA quantification. For each mature miRNA, reference and variations were then denoted as no 5′ shift, 1-3 nt upstream shift, and 1-3 nt downstream shift. The expression levels of these miRNAs were defined as the sum of the copy number of reads with the same 5′-position as these miRNAs. PCA on the basis of the quantified mature miRNAs (reads per million mapped reads, RPM) revealed two TSC-2D samples as outliers, which were removed from further analysis (CT27 and CT29). CT27-TSCs showed the lowest read counts (one-third lower than other samples), suggesting loss of miRNAs. Over 60% of the reads in CT29-TSCs were longer than 32 nt, which indicates contamination of other RNA types.

Differential expression analyses between TSC-2D and TSC-3D and between TSC-2D and TOs were conducted using DESeq2 (Love et al., 2014). Differentially expressed miRNAs were determined based on the false discovery rate (FDR <0.05) and fold change (>2). Predicted miRNAs targeting HLA-A with target score greater than 70 were obtained from miRDB (Wong and Wang, 2015), and their expression changes between TSC-2D and TSC-3D were illustrated in volcano plots using the Bioconductor package EnhancedVolcano (Blighe and Rana, 2018).

Raw sequencing reads were deposited in EMBL-EBI ArrayExpress with experimental code E-MTAB-10438.

### Statistics and reproducibility

For all experiments, multiple patient lines were used, with the specific *n* listed in the figure legends. All experiments involving TSCs had representation from both blastocyst-derived and first-trimester placenta-derived lines. Except in the case of RNA and small RNA-seq, each experiment was repeated a minimum of twice independently (technical replicates). Statistical analyses were performed by a paired two-tailed Student's *t*-test (Fig. 1J) and an unpaired two-tailed Student's *t*-test with Welch's correction (Fig. 3D). Statistical tests were performed using GraphPad PRISM software. Statistical analyses were performed by a one-sided Wilcoxon rank-sum test in Fig. 2C, Fig. 2F and Fig. S4B.

## Supplementary Material

Supplementary information
