# Supplementary material for: Characterization of primary models of human trophoblast
Source: Development. 2021 Nov 5;148(21):dev199749. doi: 10.1242/dev.199749 (PMC8602945; doi:10.1242/dev.199749)
Supplement: Supplementary information [file develop-148-199749-s1.pdf]

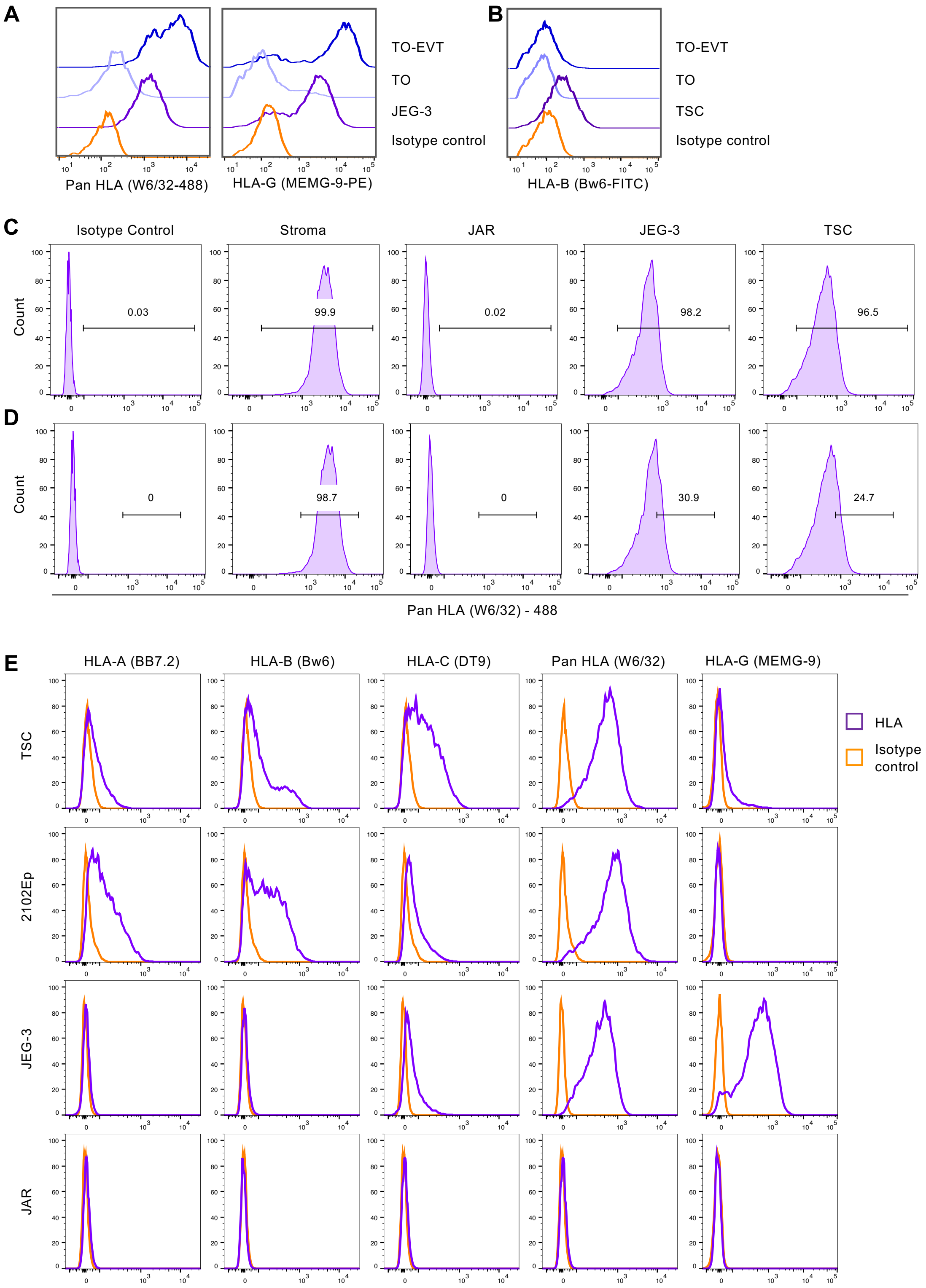
A
TO-EVT
TO
JEG-3
Isotype control
Pan HLA (W6/32-488)
HLA-G (MEMG-9-PE)
B
TO-EVT
TO
TSC
Isotype control
HLA-B (Bw6-FITC)
C
Isotype Control
Stroma
JAR
JEG-3
TSC
Count
0.03
99.9
0.02
98.2
96.5
D
Count
0
98.7
0
30.9
24.7
Pan HLA (W6/32) - 488
E
HLA-A (BB7.2)
HLA-B (Bw6)
HLA-C (DT9)
Pan HLA (W6/32)
HLA-G (MEMG-9)
TSC
2102Ep
JEG-3
JAR
HLA
Isotype control

Fig. S1. (related to Fig. 1). (A) FACS analysis of TO and TO-EVT. JEG-3 cells are the p ositive control for W6/32 and MEMG-9. TO only express W6/32 and MEMG-9 following EVT differentiation (n=4). (B) FACS analysis of TO, TO-EVT and TSC with Bw6 (HLA-B antibody). Specific lines used in this plot were DNA typed. (C) FACS analysis of stromal cells, JAR, JEG-3, and TSC with W6/32. The isotype control and JAR cells are the negative control for class I expression. JEG-3 and stromal cells are positive controls. (D) The gating strategy reported in ref (Okae *et al.*, 2018), in which stromal cells are the positive control for W6/32. JEG-3 line, which is also a positive control for class I (W6/32) are shown as mainly W6/32- if using this gating. (E) Full HLA profile of one representative TSC line (CT27) (n=4). 2102Ep is the positive control for HLA-ABC, JEG-3 is the positive control for HLA-C/G and negative control for HLA-B, respectively, and JAR is the negative control for all HLA antibodies. Isotype controls are shown in orange and the specific HLA antibodies are depicted in purple.

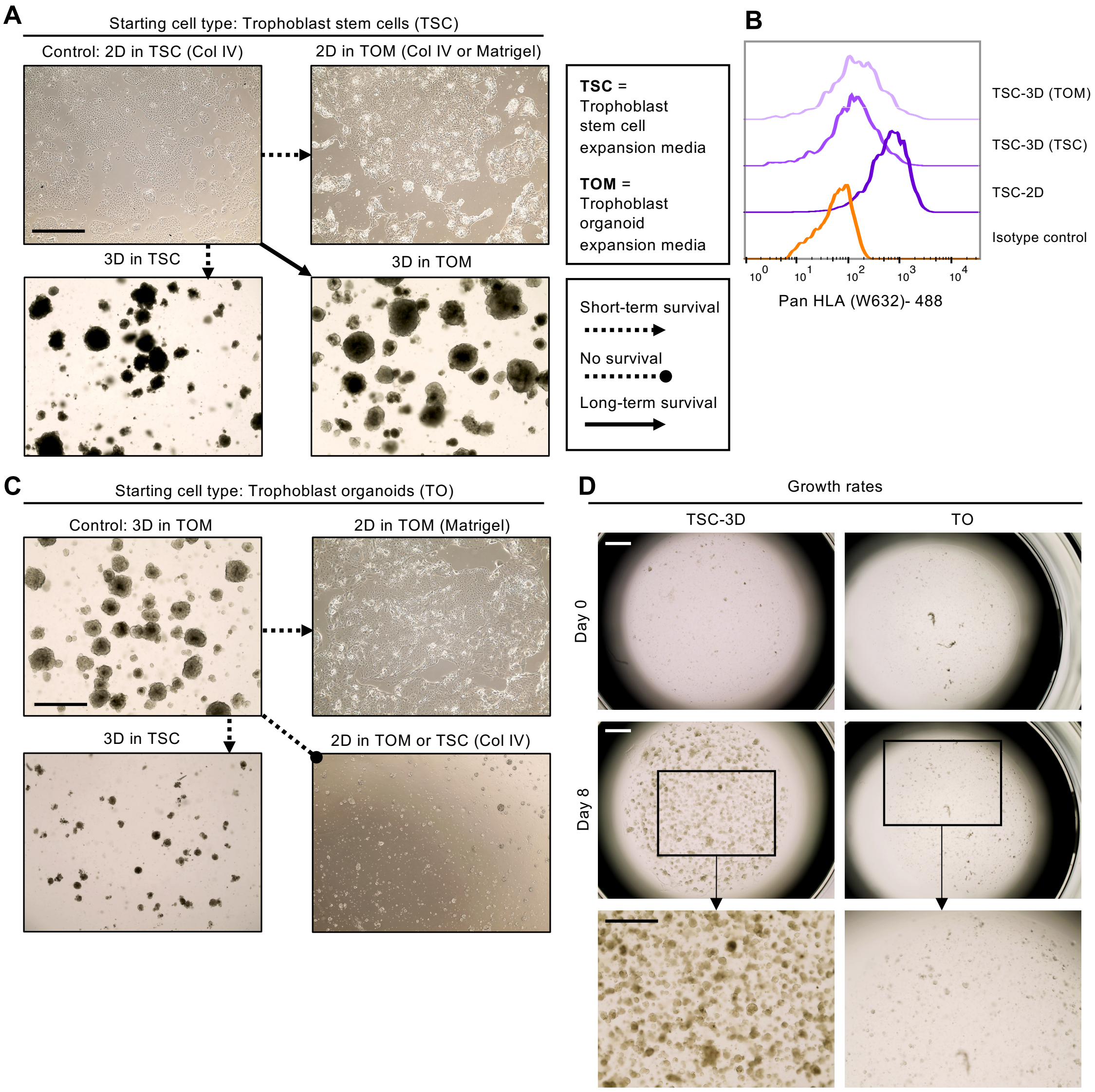

Fig. S2. (related to Fig. 1). Schematic of 2D vs 3D culturing conditions and their corresponding HLA profiles. (A) Brightfield images of the starting point (TSC, control) and the various culture conditions shown according to: plating substrate (Col IV or Matrigel), dimensions (2D or 3D) and cell culture media (TSC or TOM) used. Whether the cultures could survive or be maintained short or long term is indicated by different arrows. Short-term is defined as <10 passages (or about 2-8 weeks in culture). Scale bar is 1mm. (B) FACS analysis of TSC grown in 2D (TSC), 3D (TSC) and 3D (TOM). W6/32 expression was decreased in both 3D conditions, when compared to 2D cultures. (C) The same experimental set-up as in panel A but with TO as the starting point (control). Scale bar is 1mm. (D) Cell suspensions of TSC-3D and TO (20,000 cells/well) were plated to assess the number of organoids formed (day 0 to day 8 in culture). Scale bars are 1mm.

Development • Supplementary information

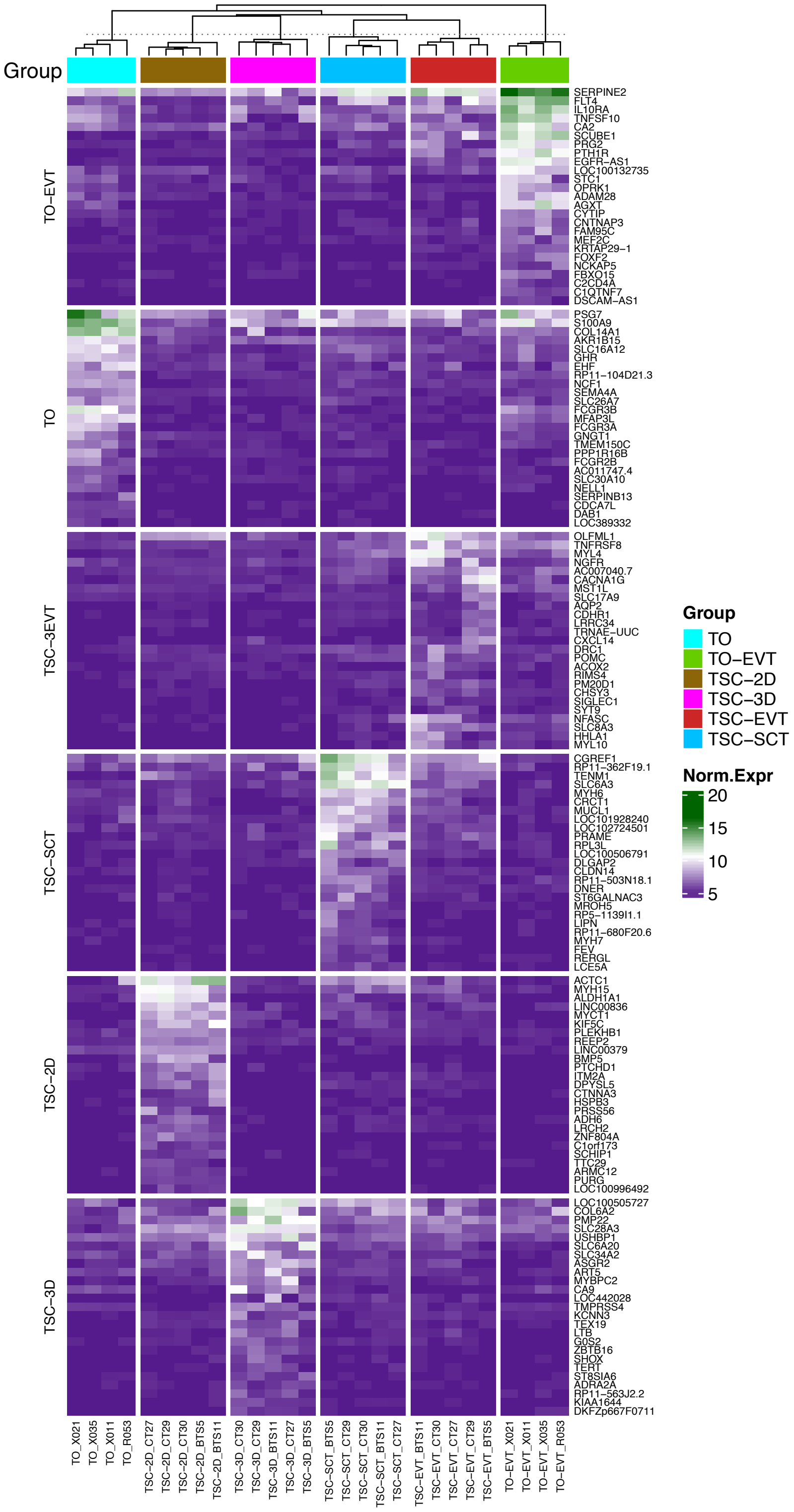

Fig. S3. (related to Fig. 2). A heatmap utilizing unbiased clustering across all six groups. One against all DESeq2 analysis was performed to determine the top up-regulated markers from each comparison. Hierarchical clustering is shown based on log2 normalized expression.

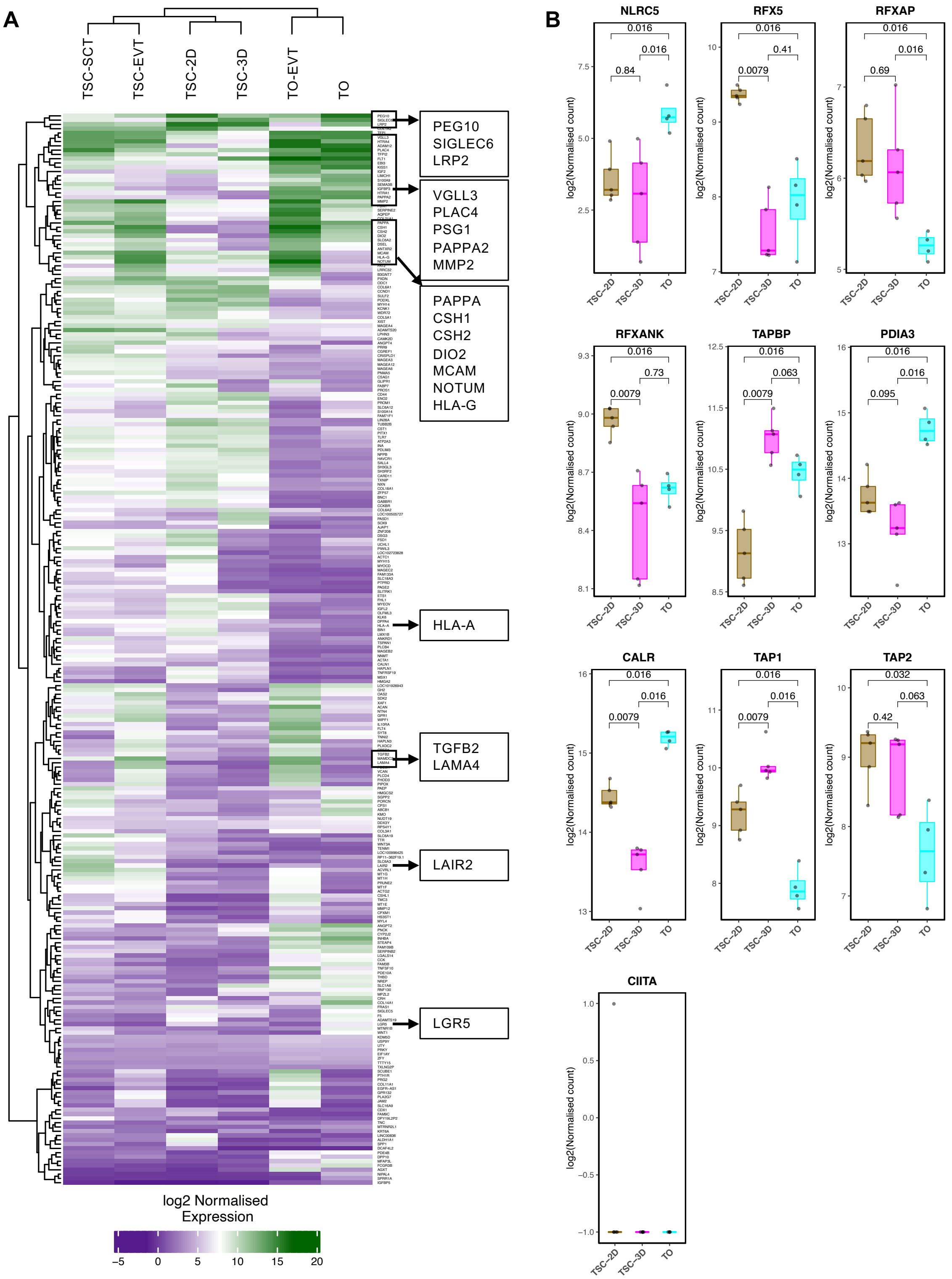

Development • Supplementary information

Fig. S4. (related to Fig. 2). (A) A heatmap of the top 250 most differentially expressed genes. Hierarchical clustering is shown based on log2 normalized expression. Genes of interest or genes that are typically associated with trophoblast are indicated with arrows/boxes. (B) Expression levels (log2 normalised counts) of genes commonly associated with HLA class I transcriptional regulation in TSC-2D, TSC-3D and TO. Pairwise comparisons were made by using a Wilcox test (specific p values are listed on the graph. Each dot corresponds to a different patient line.

Development • Supplementary information

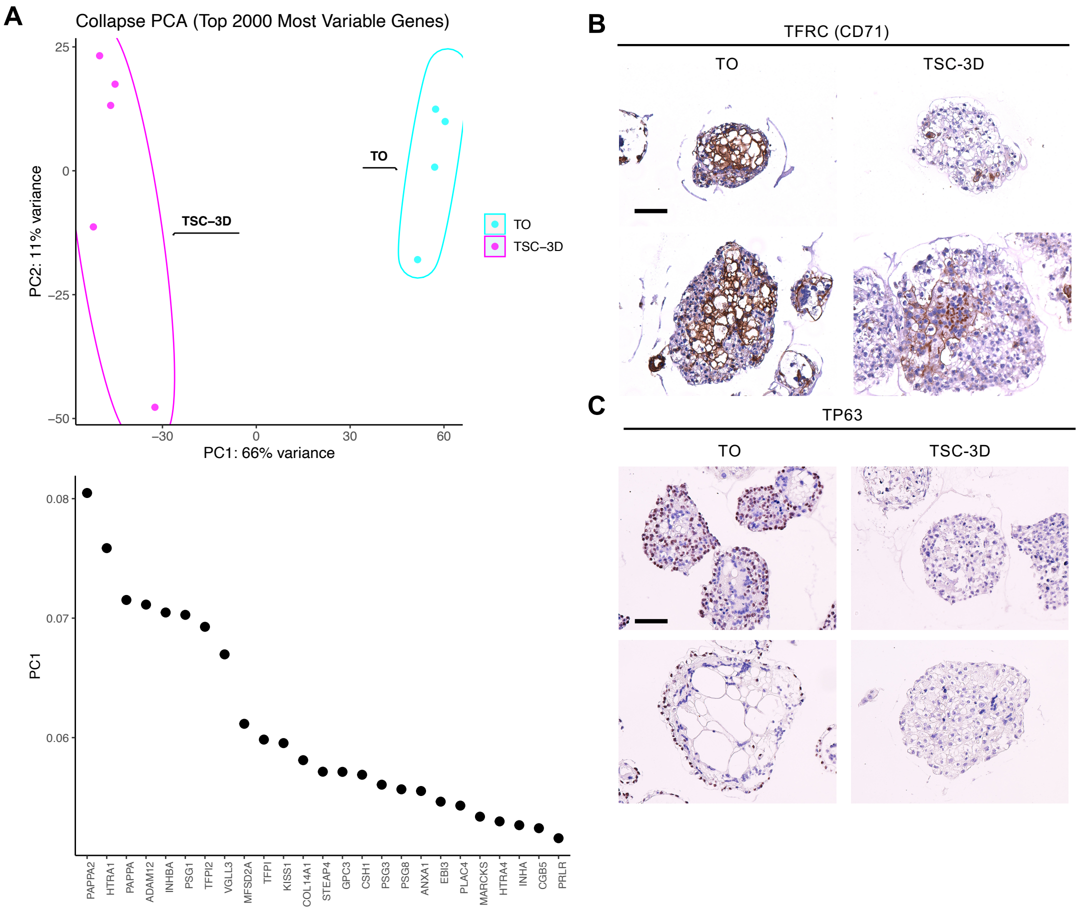

Fig. S5. (related to Figs. 2-4). (A) PCA comparing the top 2000 most variable genes between the TO and the TSC-3D. Principal component 1 (PC1) accounted for 66% of the variance and separated the two groups. Genes associated with PC1 included many syncytiotrophoblast markers (PAPPA2, PSG1, VGLL3, PAPPA, PSG3, CSH1, PSG8, PLAC4, CGB5). (B) Additional examples of IHC staining for TFRC (a syncytiotrophoblast marker) in variously sized TO and TSC-3D. Scale bar is 100 µm. (C) Additional examples of IHC staining for TP63 in variously sized TO and TSC-3D. Scale bar is 100 µm.

Table S1.

Click here to download Table S1

Table S2.

Click here to download Table S2

Table S3.

Click here to download Table S3

Table S4.

Click here to download Table S4

Table S5.

Click here to download Table S5

Table S6.

Click here to download Table S6

Table S7.

Click here to download Table S7

Table S8.

Click here to download Table S8

Table S9.

Click here to download Table S9

Table S10.

Click here to download Table S10

Table S11.

Click here to download Table S11

Table S12.

Click here to download Table S12
